# Degradation of Rural and Urban Great Tit Song: Testing Transmission Efficiency

**DOI:** 10.1371/journal.pone.0028242

**Published:** 2011-12-12

**Authors:** Emily J. Mockford, Rupert C. Marshall, Torben Dabelsteen

**Affiliations:** 1 Institute of Biological, Environmental and Rural Sciences, Aberystwyth University, Aberystwyth, United Kingdom; 2 Department of Biology, University of Copenhagen, Copenhagen, Denmark; University of Osnabrueck, Germany

## Abstract

Acoustic signals play a fundamental role in avian territory defence and mate attraction. Several studies have now shown that spectral properties of bird song differ between urban and rural environments. Previously this has been attributed to competition for acoustic space as a result of low-frequency noise present in cities. However, the physical structure of urban areas may have a contributory effect. Here we investigate the sound degradation properties of woodland and city environments using both urban and rural great tit song. We show that although urban surroundings caused significantly less degradation to both songs, the transmission efficiency of rural song compared to urban song was significantly lower in the city. While differences between the two songs in woodland were generally minimal, some measures of the transmission efficiency of rural song were significantly lower than those of urban song, suggesting additional benefits to singing rural songs in this setting. In an attempt to create artificial urban song, we mimicked the increase in minimum frequency found several times previously in urban song. However, this did not replicate the same transmission properties as true urban song, suggesting changes in other song characteristics, such as temporal adjustments, are needed to further increase transmission of an avian signal in the city. We suggest that the structure of the acoustic environment, in addition to the background noise, plays an important role in signal adaptation.

## Introduction

Acoustic signalling plays a fundamental role in avian communication [1]. In addition to begging, contact and alarm calls, in many bird species, male song has been shown to lie at the heart of territorial disputes (e.g. [2]) and mate attraction (e.g. [3]). Recent research in several countries has revealed changes in avian acoustic communication in the presence of anthropogenic factors. In studies within urban areas, a correlation between song frequency and urban noise has been found in great tits *Parus major* [4], [5], [6], house finches *Carpodacus mexicanus* [7] and song sparrows *Melospiza melodia* [8]. Other studies investigating song differences between urban and rural habitat have also found higher song frequencies in city environments in dark-eyed juncos *Junco hyernalis* [9], blackbirds *Turdus merula* [10], [11], and great tits [12], notably within their dispersal distance [13].

The implications of divergent urban song are not yet fully understood. However, there is evidence that rural and urban great tits are able to recognise the difference in frequency, responding more strongly to songs sung by males in areas with similar noise levels to their own than to males in noisier or quieter locations [13]. This behavioural variation in response to different song types has also recently been found in blackbirds, where forest birds showed a stronger response to lower frequency song motifs, whereas urban males showed a stronger response to higher frequency motifs [10].

Most studies have attributed the difference in song frequency to acoustic competition with anthropogenic noise in urban surroundings. Urban noise predominantly occupies the lower frequencies of the spectrum and, by raising the frequency of their song, male songbirds may have an opportunity to avoid introducing other costs such as increasing amplitude which in turn may increase predation [14], [15], [16], [17] or parasite risk [17], [18]. Recent experiments on wild great tits [19] and house finches [20] have shown a real-time spectral shift in response to playback of city noise: upon being subjected to an increase in low-frequency noise, both species showed an immediate increase in song frequency. In response to a playback of reversed city noise (i.e. high-frequency noise rather than low-frequency), individuals that switched did so to a lower frequency song type [19]. Therefore an instant compensatory mechanism is used to deal with high noise level, although there may also be other factors influencing the frequency choice seen in the field. Great tits are known to exhibit different song structures and frequencies according to both the density of woodland in which they live [21] and the density of individuals in a given area [6]. The variation in the acoustic structure of towns and cities and the density of individuals within them may therefore act as a contributory stimulus for the observed variation in song frequency.

All sounds degrade after leaving their source. In a completely homogenous environment, spherical spreading causes a standard attenuation of 6 dB per doubling distance around the point source of sound. However, natural environments are not homogenous and any structural fluctuation will cause further degradation to the sound signal. Sound degradation refers to any change in spectral, temporal, and structural characteristics occurring between the sender and receiver of the signal [22] and can occur by refraction, for example from a change in wind speed, reflection from structural surfaces and diffraction round objects in the environment. In addition, attenuation in excess of spherical spreading will be caused by structural objects, temperature and humidity. In any environment, small changes can have a considerable effect on the efficiency with which sounds travel and so bestow favourability upon certain songs.

The Acoustic Adaptation Hypothesis (AAH) [23], [24] describes the mechanism by which birds use the acoustic properties of the environment surrounding them to choose appropriate songs for their purpose. Many studies have found differences in songs associated with varying habitat types (e.g. [25], [26], [27]), while other studies have proved that sound degradation does differ between habitats (e.g. [28], [29], [30]). Characteristically, songs from forest habitats are lower in frequency, have a narrow frequency range and consist of long, simple notes. On the other hand, songs from open habitats are often higher in frequency, have a large frequency range and consist of more complex notes [22]. Even a small change in structure such as foliation of trees in a woodland habitat considerably increases degradation of great tit song [31].

The structural differences between rural and urban environments are extensive. Whereas rural woodland territories are usually fairly uniform and generally absorptive of sound with small reflective surfaces in the foliage, cities consist of large-scale reflective surfaces, fewer absorptive surfaces, and acoustic canyons. The high reflectance allows sound to ricochet and linger, leading to flutter-echoes which, if they arrived quickly could mask or even relocate the sound source from the receiver's point of view [32]. This effect is similar to reverberation within a woodland environment but the high level of reflection in urban surrounds means that the echo will retain a higher proportion of the original energy. It is therefore likely that the differences in sound transmission properties between the two environments are sufficient to influence the chosen song type of the bird [33]. A study into the sound propagation characteristics of urban and forest dark-eyed junco *Junco hyernalis* territories revealed differences when transmitting artificial sounds [9]. In forests, tails of reflected sounds had gradually decreasing amplitude, whereas on an urban site (a university campus), there were multiple discrete echoes. A song analysis of males occupying these sites showed a higher minimum frequency in the urban birds compared to three of the four forest sites [9]. While this study only uses one urban population, it does suggest that structurally dependent signal transmission may be influencing song characteristics.

Great tits have repeatedly been shown to sing songs at a higher average minimum frequency in urban environments than in rural environments [4], [5], [10], [11]. Great tit song consists of repeated phrases of 1–6 notes, although the most common and recognised song type is made up of two alternating notes, one high and one low. Notes can be a pure tone with a narrow frequency bandwidth, or a buzz note which has a broader bandwidth. The phrases are repeated several times, followed by a gap of a few seconds before the song is started again. Great tits are ubiquitous across the UK and, while their natural habitat is deciduous forest, they readily take to urban habitat [34]. Here we investigate (a) whether transmission of urban great tit songs is more efficient than rural songs in an urban environment, (b) whether transmission of rural great tit songs is more efficient than urban songs in a rural environment, and (c) whether creating artificial urban songs, by raising the minimum frequency of rural songs, replicates the transmission properties of urban songs in both rural and urban environments.

## Methods

### Experimental site

The experiment was conducted in Sheffield, U.K., from 14–23 February 2010. This time was chosen as it coincides with peak territory formation of this species. It is also before leaf burst, which has been shown to have a considerable effect on signal transmission [31]. All sites used were established great tit territories, where a singing male had been observed in the week before the experiment was carried out. Experiments on rural sites were carried out in Ecclesall Woods, a mixed, deciduous woodland approximately 6 km from the centre of Sheffield. Three urban and three rural sites were used and all experiments on the same site were carried out during one visit in an attempt to keep the environmental conditions consistent. Experiments on urban sites were carried out close to the centre of the city in open spaces surrounded by roads and buildings; one urban site was on the edge of parkland. It was necessary to carry out the urban experiments during the night so as to avoid noise from traffic and industry as much as possible. The rural experiments were carried out during the day from 10am. Weather conditions remained dry and similar throughout all the experiments (temperature: −3.3–6°C). The average background noise at rural and urban sites was comparable during the experiments (Rural: 40.7 dB(A), Urban: 40.9 dB(A)).

### Test sounds

The great tit songs used in this experiment were selected from an archive of high-quality great tit songs recorded across the UK for a previous study [13]. The songs were recorded within six meters of the singing male on a Marantz (Longford, Middlesex, UK) CP430 tape recorder, with a Sennheiser (Wedemark, Lower Saxony, Germany) ME67 unidirectional microphone. We selected nine ‘two-note’ songs from our database (all contributed by different males), six representing typical rural song and three representing typical urban song. For simplicity, we chose only pure note songs with no within-note frequency modulation. Each example song was digitalised (sampling rate: 22.05 kHz) and processed using AviSoft SASLAB Pro v5.1.01 (Avisoft Bioacoustics, Berlin, Germany). Two clear notes from each song were individually band-pass filtered using high and low-pass values deduced from visually inspecting the spectrogram. The inter-note intervals were preserved from the original song and the two notes were repeated to form a phrase of four notes (high, low, high, low).

**Figure 1:**
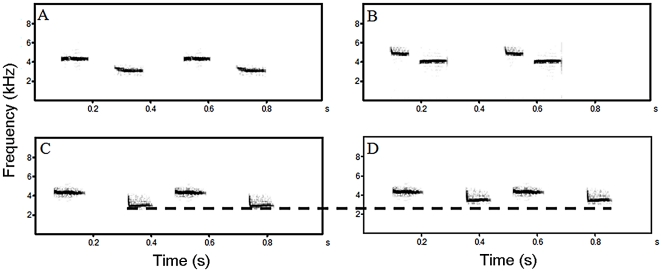
Sonograms showing example test sounds used in the experiment. Sounds A (rural) and B (true urban) were used in experiment 1. Sounds C (rural) and D (artificial urban) were used in experiment 2. The dotted line allows comparison between the frequency of the lower note in the original rural song (C) and its frequency after being increased by 500 Hz in artificial urban song (D).

Rural song types 1R–3R and urban song types 1 U–3 U were set aside for experiment one (see fig. 1A and 1B). The song frequency range (minimum frequency of low note – maximum frequency of high note) for rural songs 1R–3R was 2.4–4.6 kHz and for urban songs 1 U–3 U was 3.6–5.3 kHz. Rural song types 4R–6R were adjusted to create artificial urban song by increasing the frequency of the lower note by 500 Hz, resulting in three artificial urban song types (1A–3A). This spectral change was used as it has been found to represent the average frequency change between rural and urban songs in a previous study [13]. 4R–6R and 1A–3A were the test sounds for experiment two (see fig. 1C and 1D). The song frequency range for rural songs 4R–6R was 2.6–4.9 and for artificial urban songs (1A–3A) was 3.1–4.9 kHz.

The 12 songs were normalised to the same peak amplitude and arranged in a random order for each playback. Each was repeated ten times with one second between each repetition, and two seconds between songs of a different type to allow analysis of background noise relevant to each song.

### Field recordings

Test sounds were played back from a speaker height of six meters to microphones at a height of two and six meters from a distance of either 12 or 48 m. These heights are a realistic representation of great tit song perch height and receiver positions in woodland habitat. The distances are based on territory size found in a previous study [31], yet shortened slightly for practical reasons when carrying out the urban experiments. However, this still remains a realistic territory size [35]. Both the speaker and microphones were moved between experiments at different distances to account for small changes in habitat structure. The test sounds were also recorded at a distance of 1.5 m in an open space to control for possible effects of the equipment on the sound transmission, these recordings were used as model sounds to compare to the observation sounds recorded in the rural and urban habitats. Sounds were played from a Samsung N110 portable laptop using AviSoft SASLab Pro v5.1.01 attached to a Denon DCA-600 power-amplifier, connected to a Vifa 1'D26NC-05-06 neodymium tweeter [36]. The sounds were recorded onto a Marantz PMD671 digital recorder attached to a preamplifier (G.R.A.S. Power Module Type 12AA) using two microphones (G.R.A.S. Type 40AF) attached to individual preamplifiers (G.R.A.S. Type 26AK). Both microphones were attached to a telescopic mast, at two and six meters in height. The speaker and microphones were pointed towards each other for all experiments. The preamplifier was set to +20 dB during all observational sounds and 0 dB during the model sound recordings. This difference was accounted for in the subsequent calculations. All songs were played at a sound pressure level of 68–69 dB measured from 10 m away, with a Brüel and Kjær SPL meter (type 2236, A-filter, fast setting) [31].

### Sound analysis

By comparing the model and observational sounds, we measured sound degradation using the program SIGPRO v3.23 [37]. We followed established protocol [28], [29], [30], [31], [38] to analyse the first two observations (an observation is a four note phrase) of each song type that did not overlap with transient noise of the same frequency after digitalisation at a sampling rate of 22.05 kHz. Analysis was carried out on the second two notes to allow for effects of singing phrases in succession, the usual singing pattern of great tits. The background noise was compensated for by measuring it in a band-limited 1 s interval, close to the signal being examined.

Comparison of model and observation sounds through cross-correlation measures of the waveforms allowed us to determine the signal-to-noise ratio (SNR), the amount of energy in the observation compared to the amount of energy in the background noise and the tail-to-signal ratio (TSR) the amount of energy in the tail of the signal compared to the signal itself. Comparison of the amplitude function allowed us to measure the excess attenuation (EA), attenuation beyond that which is caused by spherical spreading, and the blur ratio (BR), the temporal distortion and frequency-dependant attenuation. Detailed procedures and formulae for each measure are outlined in Dabelsteen *et al.* [30], Holland *et al.* [38] and Balsby *et al.* [28]. After sound analysis, it was found that all EA values were less than zero, an effect of the model sounds being recorded at lower amplitude. However, as this error was present across all results, analysis was still possible as rural and urban measurements are directly comparable. In summary, a low EA, BR and TSR, and a high SNR all indicate less degradation. The time at which 75%, 50% and 25% of the tail energy remained after transmission of the original signal had ceased was also calculated according to the formulae in Holland *et al.* [39] and these quartiles were used to determine the rate of tail energy decline (RTD). Overall 1152 sounds were analysed.

### Meteorological and environmental considerations

The measures blur ratio (BR), tail-to-signal ratio (TSR) and rate of tail energy (RTD) decline all depend to a large extent on physical obstacles and reverberating surfaces which are unlikely to vary over the day, especially in the urban sites. Although dew on thin, new leaves could change the scattering ability of the leaves slightly [40], this phenomenon would only affect excess attenuation (EA) very slightly and we conducted our experiments in the winter with no such leaves on the trees.

The only measure that might be affected by climatic conditions is EA. Theoretically, clear negative or positive temperature gradients over ground level (i.e. temperature decreasing and increasing over the ground and upwards in the air, respectively) may cause sound shadows in the middle of the day and night channelling, respectively, thus affecting EA. However, these phenomena are highly unlikely in February with the temperatures we recorded during the experiment. Negative temperature gradients usually occur later in the year from April onwards in open meadows on relatively warm days, and night channelling requires hot and sunny days followed by cloud free nights where the temperature goes down quickly at the ground because of heat radiation to the atmosphere.

A previous experiment in late April in a rural site in Denmark (Dabelsteen & Mathevon 2002) failed to show any effect of time of the day on BR and TSR, and only a very small effect on EA. However, at the time of that study new leaves were present on many of the trees and temperatures were considerably higher.

Other factors, such as absorption that varies with relative humidity is also unlikely to have varied between our two experimental habitats. If the time of day were to have an effect, it would most likely be increased relative humidity in the town at night, making the night conditions in the town more similar to the day conditions in the rural sites. However, the very low variation in temperature makes it unlikely that any bias was introduced because of this factor.

Overall, it is unlikely that climatic conditions could have introduced any bias in our analysis given the temperature conditions at the time of the year and the condition of the rural sites without leaves.

### Statistical analysis

All statistical analysis was carried out in SPSS v17.0. Within each experiment (1: rural vs. true urban song, 2: rural vs. artificial urban song), the measurements for rural and urban sites were pooled and we used a 2_site type_×2_song type_×2_note_×2_microphone height_×2_distance_ factorial ANOVA as it allowed us to include all variables and interactions. We have only included two-way interactions in the analysis. To meet the requirements of ANOVA, the measures EA and BR were log transformed (base 10), and Q1 - Q3 were square root transformed, twice in the case of Q2.

## Results

### Variation in BR, EA, SNR and TSR

**Figure 2:**
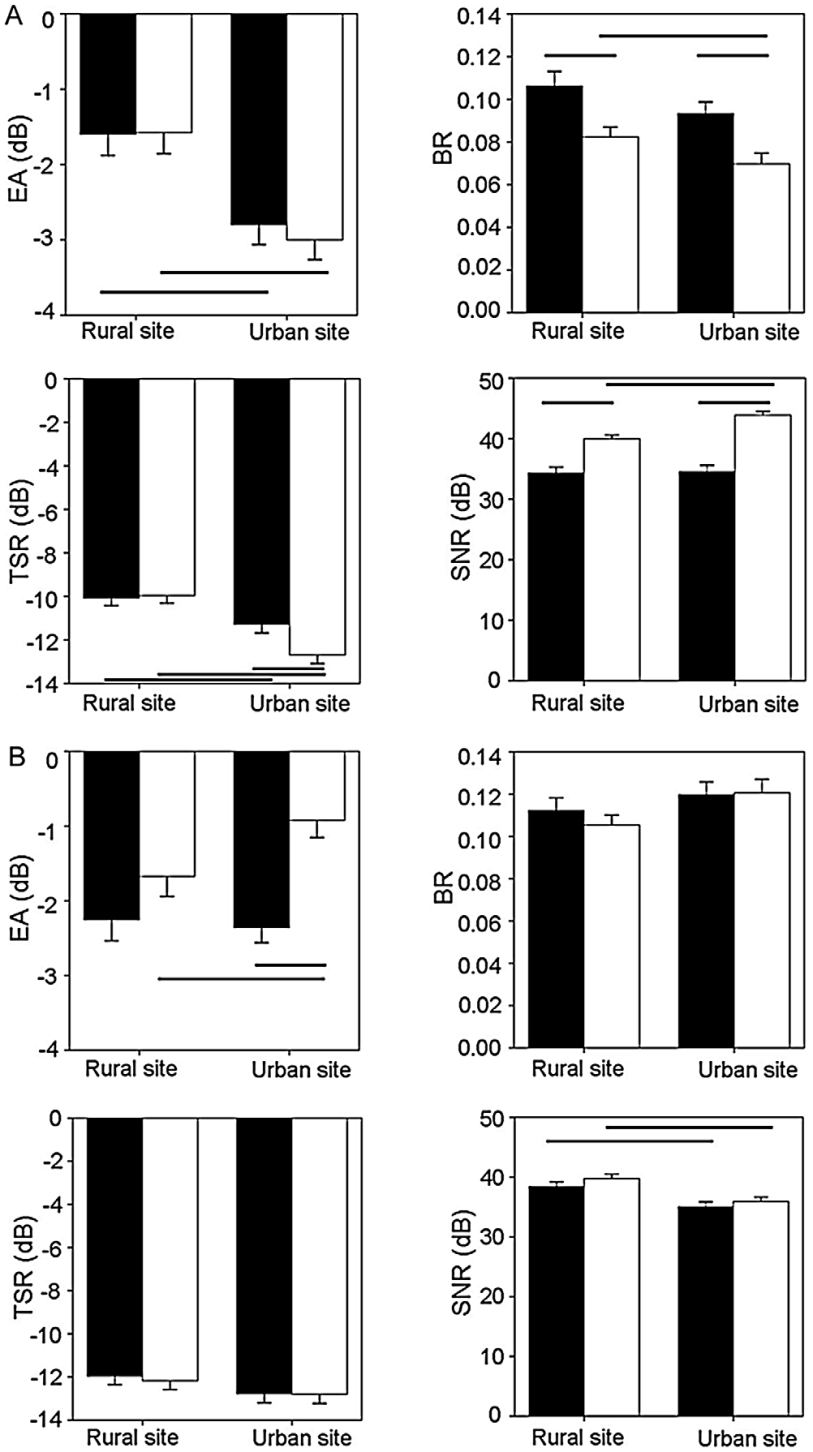
Effect of song and site on the four degradation measures. (a) experiment 1, rural and true urban song and (b) experiment 2, rural and artificial urban song. Black bars are rural song, white bars are urban song. Error bars denote one standard error of the mean. Significant differences are indicated with black lines below (EA: Excess attenuation and TSR: Tail-to-signal ratio) and above (BR: Blur ratio and SNR: Signal-to-noise ratio) the bars. The measures of EA are negative as a result of the model sounds being recorded at a lower amplitude, yet as the error was consistent across all results, rural and urban measurements are directly comparable.

In experiment one (rural vs. true urban song), site type had a significant effect on all measures of degradation, distance had an effect on all measures but EA, note had an effect on all measures except BR, and song type affected BR and SNR (table 1). Microphone height did not have any influence on any aspect of degradation (table 1). As illustrated in figure 2a, overall there was less degradation in urban habitats than rural ones for both urban (decreased BR, EA and TSR, increased SNR) and rural song (decreased EA and TSR). Compared to rural song, urban song had a consistently lower BR and higher SNR at both sites and a lower TSR at urban sites. An interaction between song and site type significantly influenced SNR and TSR.

**Table 1:** Factorial ANOVA table of experiment 1 showing the main effects and two-way interactions of all the variables on the four measurements of degradation.

	BR		EA		SNR		TSR	
	*F*	*p*	*F*	*p*	*F*	*p*	*F*	*p*
Distance	17.274	<0.001	0.490	0.484	530.582	<0.001	105.512	<0.001
Mic. height	0.346	0.557	0.157	0.692	0.306	0.580	0.340	0.560
Note	0.212	0.645	38.427	<0.001	262.833	<0.001	49.034	<0.001
Site type	9.113	0.003	28.242	<0.001	18.396	<0.001	35.523	<0.001
Song type	19.007	<0.001	0.421	0.517	241.187	<0.001	3.723	0.054
Distance×Mic. height	3.223	0.073	0.318	0.573	<0.001	0.991	0.743	0.389
Distance×note	0.008	0.928	0.502	0.479	0.129	0.719	<0.001	0.997
Distance×site type	0.608	0.436	55.455	<0.001	5.201	0.023	<0.001	0.998
Distance×song type	1.025	0.312	0.965	0.326	3.202	0.074	1.434	0.232
Mic. height×note	0.113	0.737	2.764	0.097	1.905	0.168	0.768	0.381
Mic. height×site type	0.768	0.381	3.109	0.780	0.250	0.617	0.264	0.608
Mic. height×song type	1.808	0.179	0.003	0.958	0.168	0.682	0.062	0.803
Note×site type	0.058	0.810	7.255	0.007	8.240	0.004	1.208	0.272
Note×song type	15.825	<0.001	5.154	0.024	380.802	<0.001	9.764	0.002
Site type×song type	1.491	0.223	0.508	0.476	14.589	<0.001	5.273	0.022

All d.f. = 1.

**Figure 3:**
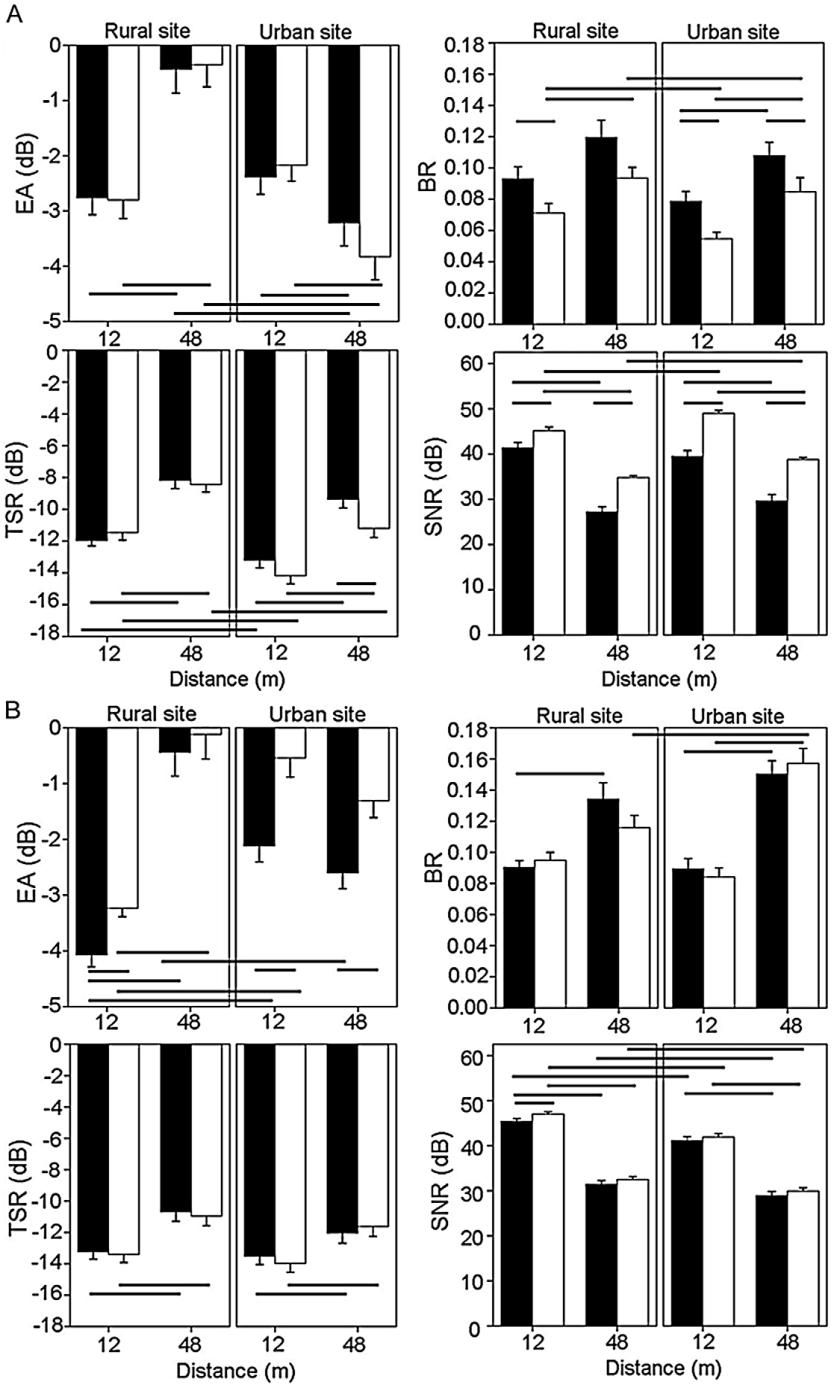
Effect of distance on the four degradation measures. (a) experiment 1, rural and true urban song and (b) experiment 2, rural and artificial urban song. Black bars are rural song, white bars are urban song. Error bars denote one standard error of the mean. Significant differences are indicated with black lines below (EA: Excess attenuation and TSR: Tail-to-signal ratio) and above (BR: Blur ratio and SNR: Signal-to-noise ratio) the bars. The measures of EA are negative as a result of the model sounds being recorded at a lower amplitude, yet as the error was consistent across all results, rural and urban measurements are directly comparable.

**Figure 4:**
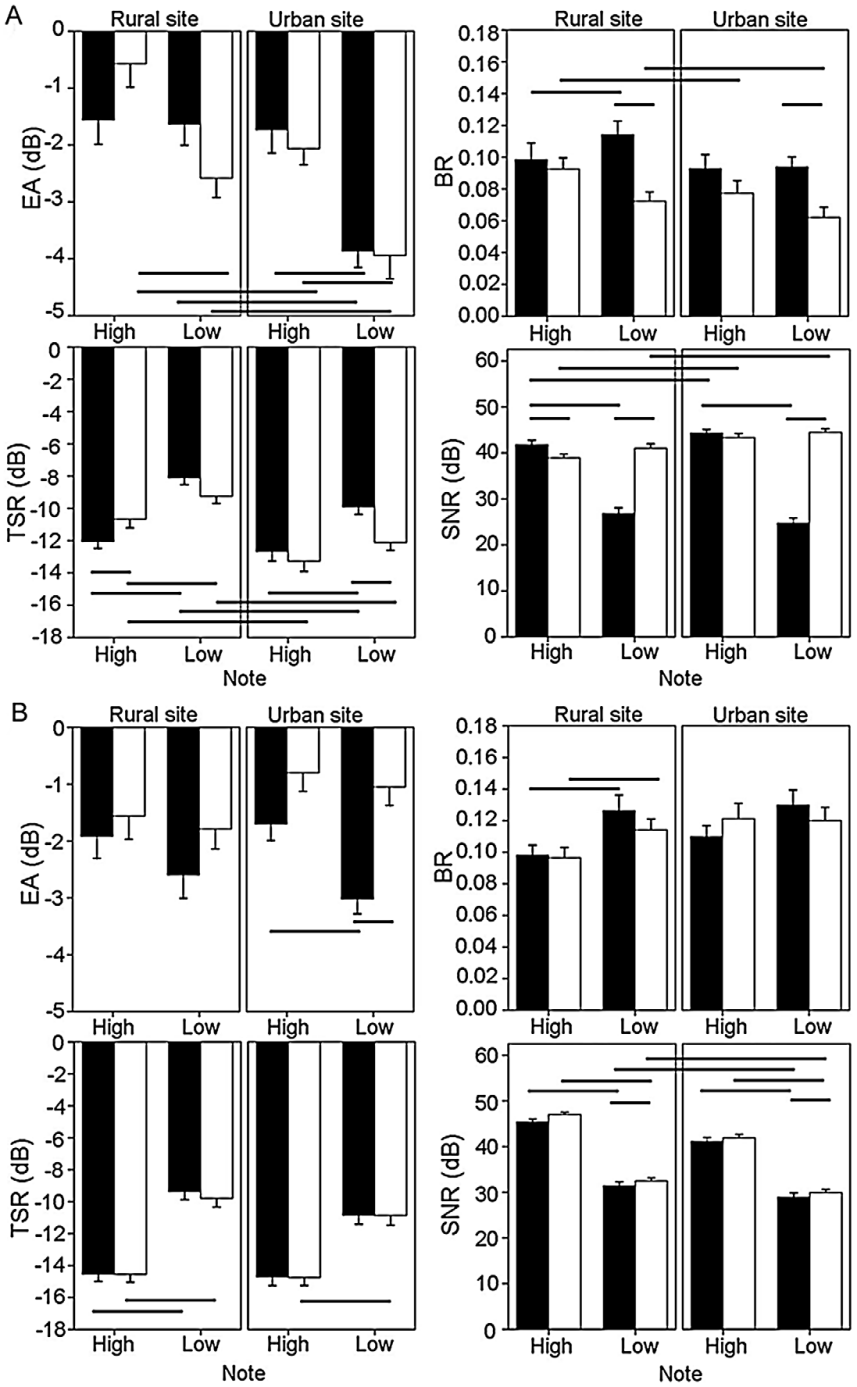
Effect of note on the four degradation measures. (a) experiment 1, rural and true urban song and (b) experiment 2, rural and artificial urban song. Black bars are rural song, white bars are urban song. Error bars denote one standard error of the mean. Significant differences are indicated with black lines below (EA: Excess attenuation and TSR: Tail-to-signal ratio) and above (BR: Blur ratio and SNR: Signal-to-noise ratio) the bars. The measures of EA are negative as a result of the model sounds being recorded at a lower amplitude, yet as the error was consistent across all results, rural and urban measurements are directly comparable.

Although distance accounted for a lot of variability across the main effects (1%–48.7%), only the interaction of distance×site type affected EA and SNR. Distance had a different effect on EA in urban habitats than rural habitats, causing a decrease instead of an increase respectively in both song types at 48 m (fig. 3a). There was no difference in EA between songs at any distance×site type combination. In all other measures, an increase in distance caused an increase in degradation in both songs (increased BR and TSR, decreased SNR). The only two-way interaction to affect all measures significantly was note×song type. The EA of the low note in both song types decreased in urban habitat (fig. 4a). The difference in SNR and TSR between the low and high note is substantially more in the rural song than the urban song in both environments. This is particularly noticeable in the SNR, where the value for the low note of the rural song is much lower in both sites types. The BR of the low note was significantly lower in urban song than rural song at both sites. Note×site type also had a significant effect on EA and SNR (table 1).

In experiment 2 (rural vs. artificial urban song), site type had an effect on EA, SNR and TSR and song type had an effect on EA and SNR. In contradiction to experiment one, microphone height had a significant effect on BR and EA (table 2). The artificial urban song displayed an increased EA when played in an urban environment, unlike the natural urban song in experiment one (fig. 2b). Neither BR nor TSR showed any change for either song between sites whereas both songs decreased in SNR in the urban environment. Both distance and note had a significant effect on all four measures. Distance explained 5.6–69.7% of the variability across the measures of degradation. Of all the two-way interactions, distance×site type affected the most degradation measures – EA, BR and SNR. As in experiment one, both songs show an increased EA at 48 m compared to 12 m in the rural habitat but an increase at 48 m in the urban habitat was not present in the urban environment in experiment two (fig. 3b). There were no differences between sites in TSR, and no interaction affected this measure. As well as a main effect, microphone height had an effect on EA and BR in conjunction with site type and distance respectively, and note×song type significantly affected EA. Unlike experiment one, where the EA of the urban song decreases for the low note in the urban site, there is no difference in experiment two (fig. 4b). SNR was also affected by note×song type, along with note×site type. The low note of both urban and rural songs had a decreased SNR compared to the high note, and when played in an urban site compared to a rural site.

**Table 2:** Factorial ANOVA table of experiment 2 showing the main effects and two-way interactions of all the variables on the four measurements of degradation.

	BR		EA		SNR		TSR	
	*F*	*p*	*F*	*p*	*F*	*p*	*F*	*p*
Distance	65.654	<0.001	33.276	<0.001	1185.74	<0.001	148.348	<0.001
Mic. height	6.191	0.013	5.942	0.015	1.051	0.306	0.543	0.462
Note	9.122	0.003	8.781	0.003	524.363	<0.001	36.843	<0.001
Site type	1.289	0.257	4.437	0.036	89.596	<0.001	3.983	0.046
Song type	0.491	0.484	23.066	<0.001	8.343	0.004	0.101	0.751
Distance×Mic. height	5.475	0.020	0.171	0.679	2.003	0.158	2.297	0.130
Distance×note	0.012	0.913	1.792	0.181	0.074	0.786	0.327	0.567
Distance×site type	13.531	<0.001	73.994	<0.001	7.970	0.005	2.228	0.136
Distance×song type	<0.001	0.984	1.477	0.225	0.051	0.821	0.069	0.793
Mic. height×note	0.286	0.593	0.084	0.772	0.537	0.464	0.003	0.956
Mic. height×site type	0.258	0.611	6.884	0.009	0.465	0.495	0.312	0.577
Mic. height×song type	0.494	0.483	0.055	0.815	0.283	0.595	0.319	0.573
Note×site type	1.666	0.197	0.424	0.515	12.466	<0.001	0.643	0.423
Note×song type	0.154	0.695	4.775	0.029	23.065	<0.001	0.279	0.598
Site type×song type	0.016	0.900	2.626	0.106	0.369	0.544	0.073	0.787

All d.f. = 1.

### Variation in RTD and tail energy quartiles

In experiment one, distance, site type and song type all had a significant effect on all three quartiles of tail energy decline (table 3). All quartiles were larger at longer distances, in urban sites and from rural song. Q1 was larger at a microphone height of 2 m and note had a significant effect on Q2 and Q3, values being higher for low notes. Two-way interactions of distance×microphone height, distance×site, note×song type and note×site type had a significant effect on various quartiles (table 3), with note×site type having an effect on all three.

**Table 3:** Factorial ANOVA table of experiment 1 showing the main effects and two-way interactions of all the variables three quartiles of tail energy decline.

	Q1		Q2		Q3	
	*F*	*p*	*F*	*p*	*F*	*p*
Distance	25.855	<0.001	32.387	<0.001	29.807	<0.001
Mic. height	6.808	0.009	3.455	0.060	0.830	0.363
Note	0.225	0.635	6.795	0.009	9.270	0.002
Site type	6.899	0.009	27.386	<0.001	47.865	<0.001
Song type	14.680	<0.001	2.145	<0.001	20.902	<0.001
Distance×Mic. height	5.655	0.018	6.971	0.009	3.477	0.063
Distance×note	0.919	0.338	0.707	0.401	0.818	0.366
Distance×site type	2.884	0.090	10.535	0.001	18.754	<0.001
Distance×song type	1.642	0.201	2.812	0.094	1.824	0.177
Mic. height×note	0.005	0.942	0.386	0.535	0.029	0.865
Mic. height×site type	0.640	0.424	1.034	0.310	1.546	0.214
Mic. height×song type	0.005	0.946	0.150	0.698	0.651	0.420
Note×site type	3.364	0.047	2.673	0.103	1.305	0.254
Note×song type	25.644	<0.001	28.250	<0.001	36.026	<0.001
Site type×song type	0.011	0.916	0.661	0.416	1.635	0.202

All d.f. = 1.

**Figure 5:**
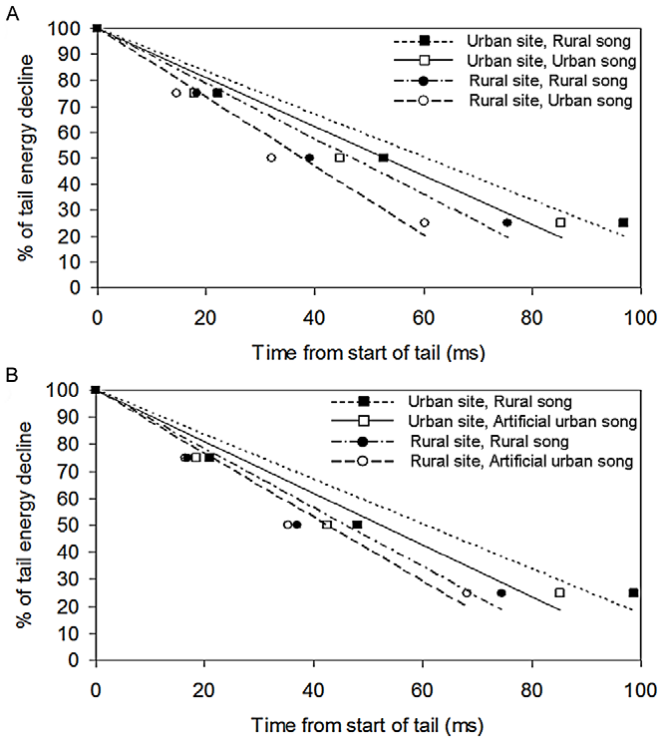
The rate of tail energy decline (RTD) for great tit songs after transmission. RTD of rural, urban & artificial urban songs, **i**ndicated by regression lines through time points 0, Q1, Q2 and Q3 (energy remaining: 100%, 75%, 50% and 25% respectively) for (a) experiment 1 and (b) experiment 2.

**Figure 6:**
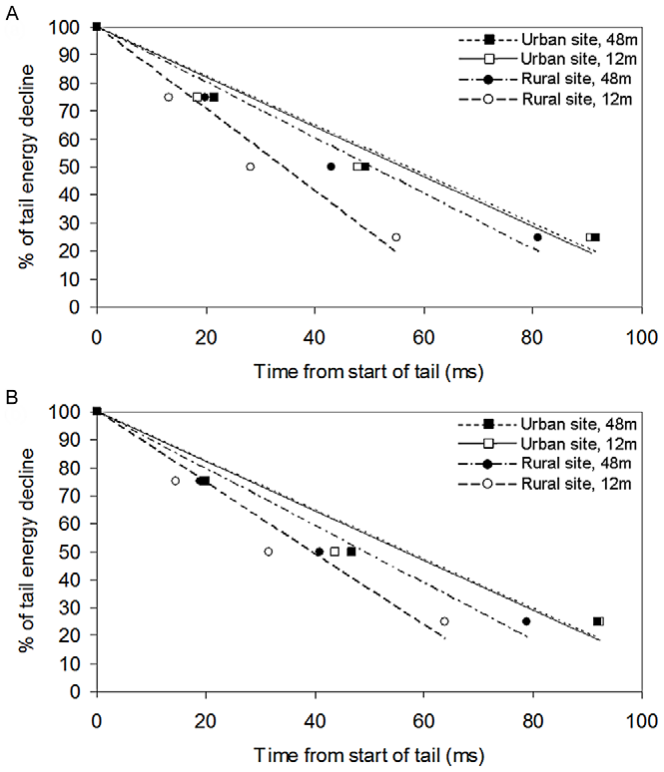
The rate of tail energy decline (RTD) for great tit songs after transmission. RTD over 12 m & 48 m, indicated by regression lines through time points 0, Q1, Q2 and Q3 (energy remaining: 100%, 75%, 50% and 25% respectively) for rural songs used in (a) experiment 1 and (b) experiment 2.

In experiment two, the only variable to affect all three quartiles was distance, all increasing at 48 m compared to 12 m (table 4). Site type affected both Q2 and Q3, both being larger in urban environments, and note and song type affected Q3 alone, where low notes and rural songs caused a larger value. The same two-way interactions which showed a significant effect in experiment 1 were also the only ones to have an effect in experiment 2, although not on as many quartiles (table 4). Both experiments showed the same pattern of tail energy decline (RTD). Urban sites had the slowest RTD, yet both true and artificial urban song had a slower RTD than rural song (fig. 5). When broken down by distance, the rural site at 12 m showed the quickest tail energy decline, while overall urban sites showed slower decline than rural sites at both distances (fig. 6).

**Table 4:** Factorial ANOVA table of experiment 2 showing the main effects and two-way interactions of all the variables three quartiles of tail energy decline.

	Q1		Q2		Q3	
	*F*	*p*	*F*	*p*	*F*	*p*
Distance	12.501	<0.001	25.669	<0.001	11.997	0.001
Mic. height	0.021	0.885	0.002	0.968	0.861	0.354
Note	1.343	0.247	3.159	0.076	15.790	<0.001
Site type	2.662	0.103	7.377	0.007	41.259	<0.001
Song type	1.644	0.200	3.172	0.075	14.171	<0.001
Distance×Mic. height	1.813	0.179	4.317	0.038	1.629	0.202
Distance×note	3.083	0.080	0.873	0.350	2.206	0.138
Distance×site type	2.056	0.152	0.838	0.360	5.685	0.017
Distance×song type	0.010	0.920	0.201	0.654	0.064	0.801
Mic. height×note	1.296	0.255	0.141	0.708	0.038	0.845
Mic. height×site type	0.588	0.443	0.648	0.421	0.027	0.868
Mic. height×song type	0.125	0.724	0.005	0.943	1.677	0.196
Note×site type	10.181	0.001	6.615	0.010	2.743	0.098
Note×song type	0.243	0.622	7.554	0.006	14.619	<0.001
Site type×song type	1.421	0.234	0.634	0.426	1.433	0.232

All d.f. = 1.

## Discussion

### Degradation of rural and true urban great tit song

Sound degradation of great tit song as it propagates through a territory is affected by the habitat of the site, the distance of the receiver, the type of song, and the pitch of the note (defined as ‘high’ or ‘low’).

The effect of the site (rural or urban) was most noticeable in the excess attenuation of both songs. In an urban setting, both songs showed a significant decrease in EA (*i.e*. increased retention of energy in the signal). This is predicted by Warren *et al.* [32], as the higher degree of reflectance in urban environments will allow sounds to retain more energy. Overall, rural environments appear to cause more degradation to both songs than urban environments. This may be due to the increased number of obstacles between the microphone and speaker, whereas in urban sites similar to the ones used in this experiment, the territories are much more open. The least degradation is seen in the urban song in the urban environment suggesting that it retains its original structure as it travels.

The Acoustic Adaptation Hypothesis (AAH) states that songs should be adapted to the sound transmission properties of the local habitat. However, the higher value of BR and lower SNR of rural song in rural environment compared to urban song does not support this and implies that there is an advantage to singing rural songs that outweighs choosing the best song for transmission. Rural great tit songs are consistently sung at lower frequencies than urban songs [12], [13]. It may be advantageous to use the degradation as distance cues in rural settings. Richards [41] first postulated that degradation of song was used as a distance cue in Carolina wrens *Thryothorus ludovicianus* and ranging cues have been specifically identified and located in wren song by Holland *et al.* [42]. Furthermore, a degraded signal has also been shown in great tits to reduce the response of a territory holder when the song is familiar, suggesting the singers of more degraded songs are perceived as being located further away [43], [44]. In woodland, it is harder to spot the signalling male in the canopy, even before leaf burst, so the degradation cues are important. In an urban environment where there are fewer trees and song posts are likely to be more visible, the emphasis may change from creating distance cues to ensuring that as much of the original signal as possible is retained, especially if the song is in competition with a higher level of background noise. A further explanation may lie in the fact that this experiment was carried out before leaf burst, and rural (lower) songs might propagate with more efficiency after this time. Lower frequencies are less affected by small obstacles in the environment [9] and therefore, if the same experiment were to be carried out after leaf burst, the urban song samples may not be as beneficial. In a study of great tit song transmission, Blumenrath and Dabelsteen [31] found that leaf burst imposes comparable degradation to doubling the distance before foliation. The frequency range of the rural songs (2.4–4.6 kHz) and the frequency range of the urban songs (3.6–5.3 kHz) give wavelength ranges of 7.2–13.8 cm and 6.3–9.2 cm, respectively. This increase in wavelength of rural song may give the signaller a transmission benefit around obstacles which outweighs the disadvantages early on in the season. However, as song is crucial in great tit territory formation [2], which in the U.K. happens before leaf burst in mid-April, this hypothesis seems less likely.

Nearly all sounds became more degraded at a distance of 48 m from the speaker than at 12 m (higher BR, TSR and lower SNR). This is expected and is in agreement with previous studies [28], [29], [30], [31]. However, the excess attenuation of the signals analysed in experiment one showed a different pattern. In a rural setting, both songs increase in EA at a greater distance, but in an urban setting, both songs showed a significantly decreased EA at 48 m compared to 12 m. If this effect is truly representative of the sound transmission, it suggests that instead of losing energy over a greater distance, both signals appear to gain energy. In an urban area where there are lots of reflective surfaces, it could be that echoes are being created and arriving at the receiver position (the microphone) at the same time as the original signal, boosting the amplitude. For this effect to occur, the echoes have to have a delay in arrival time of less than 0.5 ms [32] or the song elements have to be very long [45]. However, as this effect was not accompanied by a decrease in SNR and only relates to a decrease of 1–1.5 dB, these results should be viewed with caution. This should be looked at further using more than two different distances to achieve a more viable representation of the attenuation over these distances in urban environments, and to make sure this significant deviation was not due to local conditions experienced at the time of the experiment.

By far the most influential two-way interaction was that of note and song type. The lower note of the urban song was consistently less degraded than the lower note of the rural song, especially in urban sites. Again, the urban song is able to retain the original structure more efficiently than the rural song.

Site and song type had a highly significant effect on all three quartiles of tail energy decline. The tail energy declined most quickly in urban song at the rural site. Again, the urban site allows the signals to retain more energy for a longer amount of time, although the tails of urban song lose energy at a slightly faster rate than rural song. This may be an attempt by the singing male to reduce the lingering effect of echoes which could interfere with subsequent signals. This effect is referred to as forward masking and is noticeable in songs with a short inter-song interval for example the wren [39]. Distance had an effect on each of the tail energy quartiles, although contrary to the patterns of tail energy decline seen between song and site, it is the rural song at 12 m which loses energy the fastest. It would make sense for the shorter distances to show a high rate as there are not as many echoes and reverberations involved in a shorter transmission distance. However, the urban song at 12 m has one of the slowest RTDs, showing that even at this distance echoes are sustaining the sound for longer.

### Implications of raising the minimum frequency of rural song

The artificial urban song does not perform in the same way as true urban song; in fact there is little difference between the rural and urban songs in experiment two. This accords with some aspects of recent models of frequency manipulation in songbirds [46]. The only difference from rural song is a significantly increased EA which is accentuated in an urban environment. This is in contrast to experiment one, and suggests that raising the frequency alone is not enough to imitate true urban great tit song. As before, the artificial urban song had a significantly increased EA in urban sites compared to the original rural song. In the three other measures of degradation, true urban song degrades to a lesser degree than rural song over 48 m in urban sites (lower BR, lower TSR and a higher SNR), but not in rural sites. Here, there is only a difference in the signal-to-noise ratio at 48 m. Even though the experiments were carried out at times when background noise was at its lowest, it was not possible to eliminate it completely, therefore it may be that the urban songs have an advantage with this measure as they will be of a higher frequency than the background noise that was present. Indeed, modelling the effect of an increase in song frequency predicts only very small benefits in transmission distance for high pitched songs even in very high levels traffic noise [46]. However, in experiment two there was no difference at all between the songs in relation to BR, TSR and SNR at 12 m or 48 m suggesting once again that a single change to the frequency of the low note is not sufficient to create the acoustic properties of true urban song. All songs here showed an increase of degradation with an increase in distance.

In a stereotypical structurally urban environment, such as those used in this study with large reflective surfaces and open spaces, echoes are expected to be stronger, as shown here and also by Slabbekoorn *et al.* [9]. This would favour shorter notes, with longer intervals between them and shorter songs overall. In a study of ten urban and ten forest great tits across Europe, Slabbekoorn and den Boer-Visser [12] found this temporal divergence between the two populations as well as the previously mentioned spectral divergence: urban songs were shorter and had shorter inter-song intervals. In addition, the first note of the song was consistently shorter in urban songs compared to their rural counterparts. However, Mockford and Marshall [13] found no such temporal difference between rural and urban great tit song in the UK and a previous study comparing dark-eyed junco songs between rural and urban environments failed to find slower trill rates or shorter songs [9]. This discrepancy may be due to the specific structure of the environment. The males recorded by Mockford and Marshall [13] were classified as “urban” with respect to location and noise level, yet due to the smaller size of the cities used, there were rarely buildings above two stories in the vicinity. Small cities were chosen to allow the selection of a rural site within 3 km, the average dispersal distance of the great tit. However, as Slabbekoorn and den Boer-Visser [12] used capital cities, they were able to classify “urban” territories that were surrounded by buildings of at least four stories with vegetation cover of less than 15%. Therefore, this distinction likely led to a difference in sound transmission between the two classifications of “urban” sites and subsequently which songs would be more favourable. This shortening of temporal song characteristics could be an explanation for the difference in EA in this study as by chance, both the rural and urban songs used in experiment one contained notes which were shorter in duration than the songs used in experiment two (99.3 ms and 94.6 ms, respectively, compared to 127.8 ms) and it could also explain the differences seen between the rural song samples used in experiment 1 and 2, visible most notably on figure 3. All rates of tail energy decline in experiment two showed exactly the same pattern as in experiment one, suggesting that this aspect of degradation can be replicated by raising the minimum frequency. Slabbekoorn and den Boer-Visser's [12] finding of a shorter inter-song interval would also support the necessity for urban great tit song to have a faster RTD. The variety in note length was not controlled for and to investigate more closely, a similar experiment with controlled note lengths would have to be carried out.

Nemeth and Brumm [46] suggest that there is more than just frequency change differentiating rural and urban song. Based on models of sound transmission, they found that increasing the frequency only increased the transmission distance marginally compared to increasing the amplitude of the song and suggest that the two may be coupled; the spectral increase is a subsequent result of singing louder. While this theory has yet to be explored further, the point remains that there may be more that differentiates urban bird song from rural bird song than an increased minimum frequency. Comparisons of songs from noisy areas in rural environments with songs from a structurally urban environment may elucidate specific characteristics associated with urban noise rather than habitat. For example, chiffchaffs have recently been shown to sing at a higher frequency along noisy roads running through otherwise typical rural woodland [47].

In summary, urban song in an urban environment degrades over transmission significantly less than rural song in a rural environment, particularly by retaining energy from echoes. This may help in urban areas where the male is competing with a higher level of background noise. To a small degree, rural song also shows a decrease in degradation when sung in an urban environment but this effect is small when compared with the transmission efficiency of urban song. This has implications for birds dispersing from rural to urban areas, as lower transmission efficiency would put them at a disadvantage compared to males whose songs are adapted to the local environment. In addition, rural songs did not exhibit the most efficient sound transmission in rural territories, suggesting other benefits to singing these songs, such as providing distance cues.

Manipulating rural song by increasing the lower note of the rural song did not replicate the same degradation characteristics as true urban song in the urban area. However, an increased minimum frequency has been found in a number of species as a key difference in signal characteristics between urban and rural environments. As shown here, there may be other song differences, most likely temporal, that allow birds to sing with higher transmission efficiency in structurally urban areas. The increase of minimum frequency does increase the rate of tail energy decline, a useful trait when dealing with echoes, and does raise the note out of acoustic competition with lower frequencies, as previously suggested. However, our results suggest that the complexity of urban bird song cannot be attributed to one spectral alteration and further investigation is needed to classify how birds adapt their signals to novel acoustic and structural environments.
